# Phytochemicals, biological activity, and industrial application of lotus seedpod (*Receptaculum Nelumbinis*): A review

**DOI:** 10.3389/fnut.2022.1022794

**Published:** 2022-10-04

**Authors:** Yi-Fei Wang, Zi-Chun Shen, Jing Li, Tian Liang, Xiao-Fan Lin, Yan-Ping Li, Wei Zeng, Qi Zou, Jian-Lin Shen, Xiao-Yin Wang

**Affiliations:** ^1^School of Public Health and Health Management, Gannan Medical University, Ganzhou, China; ^2^Scientific Research Center, Gannan Medical University, Ganzhou, China; ^3^School of Basic Medical Sciences, Gannan Medical University, Ganzhou, China; ^4^Key Laboratory of Environment and Health of Ganzhou, Gannan Medical University, Ganzhou, China

**Keywords:** lotus seedpod, phytochemicals, health benefits, potential application, food by-product

## Abstract

Lotus (*Nelumbo nucifera* Gaertn.) is a well-known food and medicinal plant. Lotus seedpod (*Receptaculum Nelumbinis*) is the by-products during lotus products processing, which is considered as waste. Numerous studies have been conducted on its phytochemicals, biological activity and industrial application. However, the information on lotus seedpod is scattered and has been rarely summarized. In this review, summaries on preparation and identification of phytochemicals, the biological activities of extracts and phytochemicals, and applications of raw material, extracts and phytochemicals for lotus seedpod were made. Meanwhile, the future study trend was proposed. Recent evidence indicated that lotus seedpods extracts, obtained by non-organic and organic solvents, possessed several activities, which were influenced by extraction solvents and methods. Lotus seedpods were rich in phytochemicals categorized as different chemical groups, such as proanthocyanidins, oligomeric procyanidins, flavonoids, alkaloids, terpenoids, etc. These phytochemicals exhibited various bioactivities, including ameliorating cognitive impairment, antioxidation, antibacterial, anti-glycative, neuroprotection, anti-tyrosinase and other activities. Raw material, extracts and phytochemicals of lotus seedpods could be utilized as sources for biochar and biomass material, in food industry and as dye. This review gives well-understanding on lotus seedpod, and provides theoretical basis for its future research and application.

## Introduction

*Nelumbo nucifera* Gaertn. (also named as lotus), belonging to the mono-generic family Nelumbonaceae, is widely distributed in Asia, Americas and Oceania (1). Lotus has been cultivated as vegetable, functional food, and herb medicine for over 2,000 years (2). According to the phytomorphology, it can be divided into different parts, such as leaf, flower, stamen, rhizome, seed, seedpod and plumule (2). Almost every part can be used, and most of them are recorded in the “Chinese Pharmacopeia” (3). Among them, rhizome and seed are the main edible parts, which are popularly consumed as vegetables or functional foods due to their delicious taste and great nutritive and non-nutritive values (2).

**FIGURE 1:**
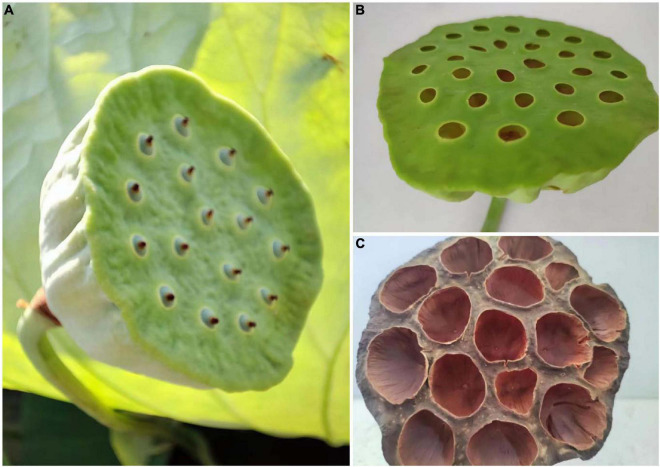
Photographs of planted lotus **(A)**, fresh lotus seedpod **(B)** and sun-dried lotus seedpod **(C)** taken by Jian-Lin Shen in August, 2022.

Lotus seedpod (*Receptaculum Nelumbinis*) is the mature receptacle of lotus house (seen in Figure 1), and is usually regarded as by-products during lotus seed processing. As one of the non-edible parts of lotus, its production is almost equivalent to the edible parts (4). In the Traditional Chinese medicine, lotus seedpod can be used for treating excessive menstrual bleeding and as hemostatic (5). According to the 2015 edition of “Chinese Pharmacopeia,” lotus seedpod charcoal (a processed product of lotus seedpod) has the usage in treating hemorrhage, urine blood, hemorrhoids bleeding, postpartum stasis, lochia, etc. However, this charcoal has gradually faded out of the market as the progress of medical science and technology. On account of lack adequate understanding on lotus seedpods, most of them are generally considered as wastes and are thrown away in the open or incinerated. This not only causes significant wastes of resources, but also brings heavy pollutions to environment. In this light, reusing the sources lotus seedpods into high value-added products is of great value.

In the past decades, more and more attention has been paid to phytochemicals, biological activity and industrial application of lotus seedpods. A variety of phytochemicals have been derived and identified from lotus seedpods, including proanthocyanidins (6), oligomeric procyanidins (7), polyphenols (8), flavonoids and others (9). Numerous studies have demonstrated that extracts and phytochemicals of lotus seedpods possessed various biological activities, such as ameliorating cognitive impairment (10–12), antioxidation (8, 9, 13, 14), antibacterial (15–17), anti-glycative (7, 18, 19) and anti-diabetes (20). Due to the good biological activities and superior physicochemical property, raw material, extracts and phytochemicals of lotus seedpods have been increasingly applied in food and other industries (21–23). Despite of multiple researches have been carried out to lotus seedpods, the information concerning it was scattered. In contrast, summary on other parts of lotus like seeds (1, 24) and leaf (25) have been reported. However, the review on lotus seedpods is rare.

Hence, in this article, the information on phytochemicals, biological activity and industrial application of lotus seedpods was systematically summarized. Meanwhile, the future study trend about lotus seedpods was proposed. This work gives well-understanding on lotus seedpod, and provides theoretical basis for research as well as exploitation and application of it, which is helpful to the protection of lotus seedpod sources.

## Preparation and identification of phytochemicals from lotus seedpods

Extracts of lotus seedpods can be mainly obtained by water (9, 14, 26–29) and organic (ethanol, methanol, glycerol, n-hexane, chloroform, ethyl acetate, butanol, acetone-water, etc.) (8, 13, 29–37) solvents. However, these traditional organic solvents methods have the limitations as low efficiency, low yield and potential environmental hazards, and water is not effective in extracting moderately polar and non-polar compounds (14). Subcritical water might be a good choice to overcome the drawback of water extraction. Anyway, water and ethanol are widely used as the extraction solvents. Chemical component analysis indicated that contents of total phenolics, flavonoids and proanthocyanidins in lotus seedpods extracts were in the range of 32.1∼607.6, 42.8∼ 862.7 and 10.6∼331.0 mg/g extract, respectively (35). In order to increase the recovery rate of phenolic compounds from lotus seedpod, ultrasound- and gas-assisted extraction methods can be applied (13, 36). Among them, the proanthocyanidins can be sequentially extracted by organic reagent like ethyl acetate to obtain the oligomeric procyanidins, which includes 10.9% catechin, 9.1% epicatechin, 53.6% dimer, 19.5% trimer and 1.9% tetramer (38, 39).

Before phytochemicals identification, the above-mentioned lotus seedpods extracts are usually purified by organic solvent (*n*-BuOH or ethyl acetate) fractionation (8, 35, 39–44), column (silica gel column, AB-8 macroporous resin or S8 resin column) adsorption (14, 35, 39, 40, 42–47), and Sephadex LH-20 column chromatography (35, 40, 46, 47). The lotus seedpods extracts before and after purification can be identified by electrospray ionization-mass spectrometry (ESI-MS) (34, 35, 46), nuclear magnetic resonance spectrometer (NMR) (34, 35, 47, 48), high-performance liquid chromatography (HPLC)/electrospray ionization tandem mass spectrometry (ESI-MS-MS) (9), HPLC-diode array detector (DAD)-ESI-MS (8), HPLC-ESI-MS (14, 49), LC-MS (38, 44), HPLC-DAD (40), HPLC-DAD-MS (50), ultra-performance liquid chromatography triple-time of flight/MS (UPLC-TOF/MS) (45, 51) and/or UPLC-quadrupole (Q) TOF-MS (41) methods. However, the analyses of phytochemicals might be limited by the capacity of the identification database, as above-mentioned detection methods depended deeply on standard secondary spectra database (41). Having considerding that used standards and the compounds in the database are limited, the completeness and accuracy of identification of phytochemicals by these methods are a little insufficient. The combination of targeted/untargeted metabolomics analysis and comparison with standards might be applied to improve this.

**FIGURE 2:**
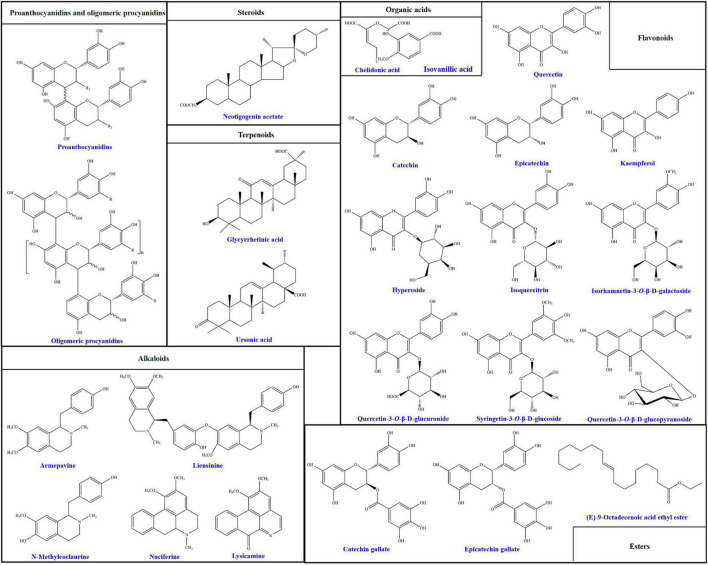
Chemical structures of some compounds identified in lotus seedpods. These chemical structures are redrawn on the basis of previous studies (8, 34, 35, 38, 39, 41, 44, 47).

Up to date, more than 94 compounds have been well-identified from lotus seedpods, as summarized in Table 1. Except the most reported two phytochemicals (proanthocyanidins and oligomeric procyanidins), the identified compounds can be generally classified into flavonoids, alkaloids, terpenoids, organic acids, steroids, esters and others (8, 14, 35, 40, 41, 45, 46). According to Table 1, the flavonoids are mostly identified in lotus seedpods. Meanwhile, Lee et al. (9) have found that the contents of 8 flavonoids included myricetin-3-galactoside, quercetin-3-glucuronide, isoquercitrin, isorhamnetin-3-glucuronide, isorhamnetin-3-glucoside, quercetin, kaempferol and isorhamnetin, were 11.52, 122.44, 29.44, 30.27, 29.73, 0.42, 2.01, and 0.80 mg/100 g respectively in lotus seedpod water extracts. Moreover, chemical structures of some compounds identified in lotus seedpods have been proposed, as illustrated in Figure 2.

**TABLE 1:** Some identified compounds from lotus seedpods *(Receptaculum Nelumbinis)*.

Class	Compound number	Name of compounds	Preparation method	Formula	*M*_w_ (Da)^α^	Identification method	References
Flavonoids	1	Hyperoside	75% ethanol extraction, *n*-BuOH fractionation, and silica gel column and Sephadex LH-20 column chromatography	C_21_H_20_O_12_	464.38	ESI-MS, ^1^H NMR and ^13^C NMR or HPLC-DAD	(35, 40)
	2	Isoquercitrin		C_21_H_20_O_12_	464.38		
	3	Quercetin-3-*O*-β-D-glucuronide		C_21_H_27_O_13_	487.44		
	4	Isorhamnetin-3-*O*-β-D-galactoside		C_21_H_17_O_13_	477.36		
	5	Syringetin-3-*O*-β-D-glucoside		C_23_H_24_O_13_	508.44		
	6	Catechin	Subcritical water extraction and AB-8 macroporous resin adsorption chromatography	C_15_H_14_O_6_	290.27	HPLC-ESI-MS^n^	(14)
	7	Cyanidin-3-*O*-glucoside		C_21_H_21_O_11_	449.39		
	8	Kaempferol-3-*O*-glucuronide		C_21_H_18_O_12_	462.37		
	9	Isorhamnetin		C_16_H_12_O_7_	316.27		
	10	Kaempferol	50% ethanol extraction and ethyl acetate fractionation	C_15_H_10_O_6_	286.24	HPLC-DAD/ESI-MS or UPLC-QTOF-MS	(8, 41)
	11	Quercetin		C_15_H_10_O_7_	302.24		
	12	Kaempferol glycosides	70% acetone extraction and S8 resin adsorption chromatography	C_21_H_20_O_10_	432.38	UPLC-TOF/MS	(45)
	13	Myricetin-3-glucoside		C_21_H_20_O_13_	480.38		
	14	Myricetin		C_15_H_10_O_8_	318.24		
	15	Rutin		C_27_H_30_O_16_	610.53		
	16	Myricetin-3-glucuronide		C_20_H_16_O_12_	448.34		
	17	Procyanidin dimer A		C_30_H_24_O_12_	576.51		
	18	Syringetin-3-glucuronide		C_23_H_22_O_14_	522.42		
	19	Quercetin glycosides		C_19_H_16_O_9_	388.33		
	20	Kaempferol-3-galctoside		C_19_H_16_O_9_	388.33		
	21	Isorhamnetin-3-neohesperidose		C_28_H_32_O_16_	624.55		
	22	Isorhamnetin-3-glucoside		C_22_H_22_O_12_	478.41		
	23	Syringetin glycosides		C_23_H_24_O_13_	508.44		
	24	Procyanidin tetramer A/B		C_51_H_54_O_31_	1162.97		
	25	Procyanidin trimmer A/B		C_36_H_36_O_21_	804.67		
	26	Procyanidin dimer A		C_30_H_24_O_13_	592.51		
	27	Cyaniding-3-galctoside		C_21_H_21_O_11_	449.39		
	28	Isorhamnetin-3-glucuronide		C_22_H_20_O_13_	492.39		
	29	Dio-7-rutinoside		C_28_H_32_O_15_	608.55		
	30	Apigenin glycosides		C_26_H_28_O_14_	564.50		
	31	Isorhamnetin 3,7-di-*O*-β-glucopyranoside	95% ethanol extraction, silica gel column and Sephadex LH-20 column chromatography	C_28_H_32_O_17_	640.55	^1^H NMR,^13^C NMR and ESI-MS	(46)
	32	(-)-Epigallocatechin		C_15_H_14_O_6_	290.27		
	33	Procyanidin B2		C_30_H_26_O_12_	578.53		
	34	2,3-*trans*-Dihydromorin		C_15_H_12_O_7_	304.26		
	35	Kaempferol-3-*O*-rutinoside		C_27_H_30_O_15_	594.53		
	36	Patuletin-7-*O*-[6″-(2-Methylbutyryl)]-glucopyranoside	80% ethanol extraction	C_26_H_28_O_14_	564.50	UPLC-QTOF-MS	(41)
	37	Apiin		C_26_H_28_O_14_	564.50		
	38	Isoetin-7-*O*-β-D-glucopyranosyl-2′-*O*-β-D-xyloypyranoside		C_26_H_28_O_16_	596.50		
	39	Taxifolin		C_15_H_12_O_7_	304.26		
	40	Isorhamnetin 3-*O*-rutinoside		C_28_H_32_O_16_	624.55		
	41	Cosmosiin		C_21_H_20_O_10_	432.38		
	42	Apigenin		C_15_H_10_O_5_	270.24		
	43	Nepitrin		C_22_H_22_O_12_	478.41		
	44	Quercetin-3-*O*-β-D-glucuronide-6″-methyl ester		C_22_H_20_O_13_	492.39		
	45	Patuletin		C_16_H_12_O_8_	332.27		
	46	Luteolin		C_15_H_10_O_6_	286.24		
	47	Morin		C_15_H_10_O_7_	302.24		
	48	Tricin		C_17_H_14_O_7_	330.29		
Alkaloids	49	Armepavine	80% ethanol extraction	C_19_H_23_NO_3_	313.40	UPLC-QTOF-MS	(41)
	50	Liensinine		C_37_H_42_N_2_O_6_	610.75		
	51	*N*-Methylcoclaurine		C_18_H_21_NO_3_	299.37		
	52	Pseudopurpurin		C_15_H_8_O_7_	300.22		
	53	Nuciferine		C_19_H_21_NO_2_	295.38		
	54	Tetrandrine		C_38_H_42_N_2_O_6_	622.76		
	55	Lysicamine		C_18_H_13_NO_3_	291.31		
	56	Morphine		C_17_H_19_NO_3_	285.34		
Terpenoids	57	Ursolic acid	95% ethanol extraction, silica gel column and Sephadex LH-20 column chromatography	C_29_H_50_O	414.71	^1^H NMR,^13^C NMR and ESI-MS	(46)
	58	Hederagenin		C_30_H_48_O_4_	472.71		
	59	Ursonic acid	80% ethanol extraction	C_30_H_46_O_3_	454.69	UPLC-QTOF-MS	(41)
	60	Glycyrrhetinic acid		C_30_H_46_O_4_	470.69		
	61	Ganoderic acid H		C_32_H_44_O_9_	572.70		
	62	Ganoderic acid G		C_30_H_44_O_8_	532.67		
	63	Soyasapogenol B		C_30_H_50_O_3_	458.72		
	64	Ganoderiol F		C_30_H_46_O_3_	454.69		
	65	Ceanothic acid		C_30_H_46_O_5_	486.69		
	66	3-*O*-β-D-Glucopyranosyl-dammar-3β,12β,20R,25-tetraol		C_36_H_64_O_9_	640.90		
	67	Pseudo-ginsenoside RT4		C_36_H_62_O_10_	654.88		
Organic acids	68	Palmitic acid	95% ethanol extraction, silica gel column and Sephadex LH-20 column chromatography	C_16_H_32_O_2_	256.43	^1^H NMR,^13^C NMR and ESI-MS	(46)
	69	Citric acid	80% ethanol extraction	C_6_H_8_O_7_	192.12	UPLC-QTOF-MS	(41)
	70	Chelidonic acid		C_7_H_4_O_6_	184.10		
	71	*p*-Coumaric acid		C_9_H_8_O_3_	164.16		
	72	Isovanillic acid		C_8_H_8_O_4_	168.15		
	73	Phellibaumin A		C_19_H_12_O_7_	352.30		
	74	Sanleng acid		C_18_H_34_O_5_	330.46		
	75	Ricinoleic acid		C_18_H_34_O_3_	298.47		
	76	Linolenic acid		C_18_H_30_O_2_	278.43		
	77	Non-adecanoic acid		C_19_H_38_O_2_	298.51		
	78	Erucic acid		C_22_H_42_O_2_	338.57		
	79	Heneicosanoic acid		C_21_H_42_O_2_	326.56		
Steroids	80	Neotigogenin acetate	80% ethanol extraction	C_29_H_46_O_4_	458.68	UPLC-QTOF-MS	(41)
	81	Daucosterol		C_35_H_60_O_6_	576.86		
	82	24-Methylenecholesterol		C_28_H_46_O	398.67		
	83	Corbisterol		C_29_H_46_O	410.68		
	84	Stigmast-4-ene-3,6-dione		C_29_H_46_O_2_	426.68		
Esters	85	Ethyl hexadecanoate	80% ethanol extraction	C_18_H_36_O_2_	284.48	UPLC-QTOF-MS	(41)
	86	Diisobutyl phthalate		C_16_H_22_O_4_	278.35		
Others	87	Hexadecan-2-ol	95% ethanol extraction, silica gel column and Sephadex LH-20 column chromatography	C_16_H_34_O	242.44	^1^H NMR,^13^C NMR and ESI-MS	(46)
	88	Pentadecan-3-ol		C_15_H_32_O	228.42		
	89	Pentadecane		C_15_H_32_	212.42		
	90	Futoamide	80% ethanol extraction	C_18_H_23_NO_3_	301.39	UPLC-QTOF-MS	(41)
	91	Cireneol G		C_17_H_30_O_2_	266.42		
	92	Isoquinolinecarboxi- midamide,3,4-dihydro-*N*-3,6,9,12,15,18,21,24-octaoxapentacos-1-yl-		C_27_H_47_N_3_O_8_	541.69		
	93	*N*-Isobutyl-2E,4E-octadecadienamide		C_22_H_41_NO	335.57		
	94	Pheophytin a		C_55_H_74_N_4_O_5_	871.22		

^α^*M*_w_ was calculated on the basis of C, H, O, and N atomic mass as 12.011, 1.00794, 15.9994, and 14.006747, respectively.

Different preparation methods produced differences in the chemical structures of phytochemicals from lotus seedpods. In the study taken by Wu et al. (35), five compounds have been respectively obtained from *n*-BuOH extracts of lotus seedpods by silica gel column chromatography with different CH_2_Cl_2_-MeOH elution ratios and Sephadex LH-20 column chromatography. These five compounds have different chemical structures, which have been identified to be hyperoside, isoquercitrin, quercetin-3-*O*-β-D-glucuronide, isorhamnetin-3-*O*-β-D-galactoside and syringetin-3-*O*-β-D-glucoside. On the other hand, extracts of lotus seedpods from different production places have been found to contain different chemical compositions. Another study of Wu et al. (40) has indicated that 50% ethanols of 20 lotus seedpods samples, collected from different regions (Fujian, Jiangxi, Zhejiang, Beijing, Jiangsu, Hubei, Hebei, and Hebei) of China, showed different contents of these five compounds. An investigation conducted by Liu et al. (50) has demonstrated that hyperoside and isoquercitrin in methanol extracts of 11 lotus seedpods samples, from different localities of Jianning County, Fujian province, China, exhibited different chemical fingerprints. Otherwise, different sources of lotus seedpods also contained different chemical compositions. The research of Limwachiranon et al. (45) has implied that lotus seedpods from three commonly consumed cultivars (Shilihehua, Jianlian and liyebailian) showed differences in phenolic, flavonoid, and proanthocyanidins contents.

## Biological activity of lotus seedpods extracts

**FIGURE 3:**
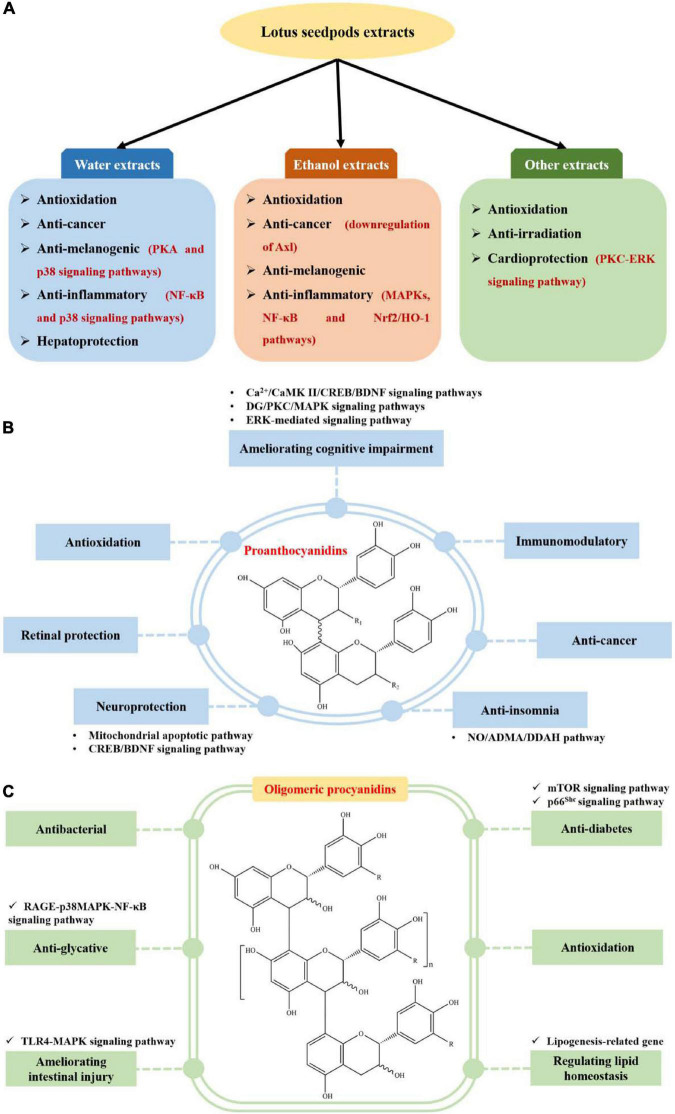
Biological activities of extracts **(A)**, proanthocyanidins **(B)**, and oligomeric procyanidin **(C)** from lotus seedpods.

Lotus seedpods extracts have been demonstrated to possess antioxidation (8, 9, 13, 14, 29, 34–36), anti-cancer (8, 14, 32), anti-melanogenic (27, 33), anti-inflammatory (28, 31), anti-irradiation (37), cardioprotection (30) and hepatoprotection (26) bioactivities, as displayed in Figure 3A and Supplementary Table 1.

### Biological activity of water extracts from lotus seedpods

Water extracts from lotus seedpods have been proven to exert antioxidation (9, 14, 29), anti-cancer (14), anti-melanogenic (27), anti-inflammatory (28) and hepatoprotection (26) activities.

In terms of the antioxidation activity, chemical assays have indicated that water extracts from lotus seedpods had scavenging effects on DPPH, ABTS and NO_2_^–^ radicals, and ferric reducing ability (14, 29). A cell experiment has demonstrated that the extracts could dose-dependently improved the survival and function of rat pancreatic RIN-m5F cells induced by H_2_O_2_ through down-regulation of apoptosis and up-regulation of autophagy, revealing as increasing protein expressions of LC3II, Atg5/12, p62, class III PI3K, Beclin-1 and p-Bad/Bad, and decreasing protein expressions of active-caspase-3 and cleavage PARP-1 along with Bax/Bcl-2 ratio (9). Regarding to anti-melanogenic effect, water extracts of lotus seedpods could inhibit melanin synthesis in α-melanocyte stimulating hormone-induced B16F10 cells and in UVB-induced mice, which involved both PKA and p38 signaling pathways (27). In the cell experiment, the extracts decreased protein expressions of p-PKA/PKA, p-p38/p38, p-CREB/CREB and MITF, and protein and mRNA expressions of tyrosinase, TRP-1 and MC1R of B16F10 cells. In the animal experiment, the extracts reduced protein expressions of tyrosinase, TRP-1, TRP-2, p-PKA/PKA and p-p38/p38 in ears of mice. As to anti-inflammatory action, water extracts of lotus seedpods exerted protective effects against LPS-induced HepG2 cells and LPS-induced mice via NF-κB and p38 signaling pathways (28). In the cell experiment, the extracts could lessen mRNA expressions of IL-6, COX-2 and iNOS, and protein expressions of COX-2, iNOS, NF-κB, IKK, p-IκB/IκB, p-p38/p38, TLR4 and MyD88 of HepG2 cells. In the animal experiment, the extracts could decrease protein expressions of COX-2, iNOS, NF-κB, IKK, p-IκB/IκB and p-p38/p38 in liver of mice. To hepatoprotection effect, water extracts of lotus seedpods reduced lipid accumulation and lipotoxicity in oleic acid-induced HepG2 cells through anti-apoptotic and anti-autophagy mechanisms, showing as adding Bcl-2 protein expression, and lowering protein expressions of LC3-II/LC3-I, Atg5/12, active-caspase-3/8/9, cleaved PARP, Bax and mitochondrial membrane depolarization, as well as Bax/Bcl-2 ratio (26). Additionally, water extract from lotus seedpods had anti-proliferation effect on HepG2 cells (14).

On the other hand, subcritical water extracts from lotus seedpods have been determined to reveal antioxidation activity, showing as scavenging DPPH, ABTS and NO_2_^–^ radicals and ferric reducing ability, and anti-cancer effect against cell proliferation of HepG2 cells (14).

### Biological activity of ethanol extracts from lotus seedpods

Ethanol extracts from lotus seedpods have been indicated to exhibit antioxidation (8, 29, 34, 35), anti-cancer (8, 32), anti-melanogenic (33) and anti-inflammatory (31) actions.

With regard to antioxidation effect, chemical assays have demonstrated that ethanol extracts from lotus seedpods exhibited scavenging effects on DPPH, ABTS, OH, O^2–^ and H_2_O_2_ radicals, ferric reducing ability and metal ion chelating activity (8, 29, 34, 35). A cell experiment has shown that the ethanol extracts revealed cytoprotection on H_2_O_2_-induced RAW264.7 cells and reduced MDA level (34). In terms of anti-cancer activity, ethanol extracts from lotus seedpods exerted anti-proliferation effect on HepG2, LNcap, A549 and H460 cells (8, 32). Thereinto, the extracts induced cell apoptosis of A549 and H460 cells via downregulation of Axl, illustrating as increase of cleavage PARP and γ-H2AX protein expressions, and decrease of PARP protein expression as well as Axl protein and mRNA expressions (32). Regarding to anti-melanogenic effect, inhibition of the extract on melanin synthesis in α-melanocyte stimulating hormone-induced B16F10 cells was related to downregulation of activity and protein expression of tyrosinase (33). As to anti-inflammatory action, MAPKs, NF-κB and Nrf2/HO-1 pathways were regulated by the extracts to LPS-induced RAW264.7 cells, which showing as increasing protein expressions of Nrf2 and HO-1, and decreasing protein expressions of iNOS, COX-2, p-p38/p38, p-ERK/ERK, p-JNK/JNK, p65 and p-p65 (31).

### Biological activity of other extracts from lotus seedpods

Methanol, glycerol, n-hexane, chloroform, ethyl acetate, butanol and acetone-water extracts from lotus seedpods have been shown to possess antioxidation (13, 29, 36), anti-irradiation (37) and cardioprotection effects (30). The extracts obtained by these solvents (except acetone-water) exerted antioxidation activities in scavenging DPPH and ABTS radicals and ferric reducing ability (13, 29, 36). Acetone-water extract of lotus seedpods showed anti-irradiation effect against ^60^Co irradiation-induced mice, behaving as increments of survival time, activities of SOD, CAT and GPX in liver, levels of white blood cells, red blood cells, platelets and hemoglobin, and spleen weight and index, and reduction of LPO level in liver and chromosomal aberrations in the bone marrow, to mice (37). 100% methanol extract had cardioprotection effect on Ang II-induced H9c2 cells through suppression of PKC-ERK signaling pathways, which exhibiting as declinations of protein expressions of NFATc-1, ANP, BNP, MLC2, NOX2, NOX4, p-NF-κB/NF-κB, AT1R, RAGE, PKC, p-PKC and p-ERK1/2 (30).

### Comparison on biological activities of different extracts from lotus seedpods

Extracts from lotus seedpods acquired with different solvents and methods had distinctions in biological activities. Antioxidation activities of 80% ethanol, *n*-hexane, chloroform, ethyl acetate, butanol and water extracts from lotus seedpods have been investigated in the study of Kim and Shin (29). The results suggested that these extracts had different flavonoid and proanthocyanidin contents, and the scavenging activities on DPPH and ABTS radicals and ferric reducing ability for them were 94.319, 33.387, 85.263, 76.099, 93.944% and 94.587, 92.937, 9.781, 93.940, 85.755, 93.184% and 93.184%, and 1.551, 0.410, 0.905, 1.099, 1.431, and 1.448 respectively, at the concentration of 0.8 mg/mL. The findings of Yan et al. (14) have manifested that subcritical water extract (SWE) and hot water extract (HWE) from lotus seedpods contained different polyphenol (815.4 and 785.6 mg GAE/g DW, respectively) and flavonoid (1012.05 and 932.56 mg RE/g DW) contents. The SWE showed significantly higher scavenging activities of DPPH, ABTS and NO_2_^–^ radicals and ferric reducing ability, along with antiproliferative activity on HepG2 cells, as compared to the HWE.

Moreover, the study taken by Bao et al. (36) has made comparisons on the antioxidant activities of extracts from lotus seedpods by four extraction means, including ultrasonic coupled with glycerol (UG), ultrasonic using water (UW), water bath incubation with glycerol (WG) and water bath incubation using water (WW). The results displayed that the extract gained by UG had the relatively highest scavenging activities on DPPH and ABTS radicals and ferric reducing ability. Another investigation of them implied that extract from lotus seedpods obtained by gas-assisted combined with glycerol approach exhibited obvious higher scavenging effets on DPPH and ABTS radicals along with ferric reducing ability than that acquired by WG (13).

## Biological activity of phytochemicals from lotus seedpods

Phytochemicals from lotus seedpods have been determined to reveal a variety of biological activities, including ameliorating cognitive impairment (10–12, 52–61), antioxidation (4, 5, 62–64), antibacterial (15–17, 65), anti-glycative (7, 18, 19), neuroprotection (48, 66), anti-tyrosinase (46, 47), retinal protection (6), anti-insomnia (67), anti-cancer (68), immunomodulatory (69), ameliorating intestinal injury (70), anti-diabetes (20), regulating lipid homeostasis (38), anti-inflammatory (71) and α-glucosidase inhibitory (4) activities.

### Biological activity of proanthocyanidins from lotus seedpods

Recently, proanthocyanidin is one of the mostly investigated phytochemicals from lotus seedpods. They have been implied to possess ameliorating cognitive impairment (11, 12, 52–61), antioxidation (62–64), neuroprotection (10, 48, 66), retinal protection (6), anti-insomnia (67), anti-cancer (68) and immunomodulatory (69) activities, as displayed in Figure 3B and Supplementary Table 1.

Animal studies have indicated that proanthocyanidins from lotus seedpods ameliorated cognitive impairment of D-galactose- (12), extremely low frequency electromagnetic fields- (11, 56, 57), scopolamine- (52–55) or alcohol-induced mice (52), senescence-accelerated mice (61), and aged rats (58–60). In terms of D-galactose-induced mice model, the ameliorating cognitive impairment action of the proanthocyanidins correlated with reverse of oxidative damage, prevention of Aβ overproduction and suppression of NO production, appearing as enhancements of SOD and GPX activities in brain, reductions of Aβ_1–42_, NO and MDA levels and AchE, MAO-B, tNOS and nNOS activities in brain, and declinations of nerve cell apoptosis and p53 protein expression in hippocampus (12). Regarding to extremely low frequency electromagnetic fields-induced mice model, Ca^2+^/CaMK II/CREB/BDNF and DG/PKC/MAPK signaling pathways involved in the ameliorating cognitive impairment effect of the proanthocyanidins (11, 57). The proanthocyanidins increased protein expressions of CaMKII, PKCα, BDNF and p-ERK1/2, and decreased concentrations of Ca^2+^, IP3, DAG, glutamate, GABA and [Ca^2+^]_*i*_ and protein expressions of Gi, PKA, PKCβ, PP2B, ASK1, NR2B, p-CREB and p-JNK1/2, in hippocampus of mice. Moreover, the action was also related to improvement of antioxidant status, showing as increasing activities of SOD, CAT and GPX, and decreasing levels of MDA and NO and activity of NOS, in serum and hippocampus of mice (56). As to scopolamine- or alcohol-induced mice model, the ameliorating cognitive impairment action of the proanthocyanidins was correalted with improvement of antioxidant ability and cholinergic activity, exhibiting as elevating T-AOC level and activities of SOD and GPX, lessening levels of MDA, MAO-B, AchE and NO, activities of MPO, AchE, tNOS, nNOS and iNOS, and nNOS mRNA expression, in hippocampus, brain, serum and/or colon (52–55). For senescence-accelerated mice model, the ameliorating cognitive impairment effect of the proanthocyanidins was in connection with boost of antioxidant level, reflecting as enhancing GSH level and SOD and GPX activities, and reducing NO and MDA levels and nNOS and total NOS activities in brain and/or serum (61). To aged rats model, the ameliorating cognitive impairment effect of the proanthocyanidins correlated with changes of NO system (60), activation of hippocampal CREB through ERK-mediated signaling pathway (59) and rejuvenation of antioxidant and cholinergic systems (58). The proanthocyanidins elevated levels of GSH, T-AOC, AchE and Ach, activities of CAT and GPX, protein expressions of p-CREB, BDNF, p-ERK42/ERK42, p-ERK44/ERK44 and iNOS, and mRNA expressions of iNOS and BDNF, and declined levels of NO and MDA and activities of tNOS and iNOS in hippocampus and/or cerebral cortex of rats.

A chemical assay has shown that proanthocyanidins of lotus seedpods exhibited antioxidation activity in scavenging DPPH and ABTS radicals along with ferric reducing ability (62). A further cell experiment has suggested that the proanthocyanidins could relieve oxidative damage in H_2_O_2_-induced HUVECs, appearing as increasing activities of SOD and GPX and production of NO, and decreasing levels of MDA and ET-1 (62). Animal experiments have proven that the proanthocyanidins exerted antioxidation action in aged rats (64) and extremely low frequency electromagnetic fields-induced mice (63). In terms of aged rats model, the proanthocyanidins promoted the activities of SOD, CAT and GPX and level of GSH, and lessened TBARS content, in serum, heart, liver, kidney, lung or muscle (64). Regarding to extremely low frequency electromagnetic fields-induced mice model, the proanthocyanidins aggrandized activities of SOD, CAT, GPX, GR and GST, and lowered MDA level, in serum and cerebral cortex (63).

Cell experiments have revealed that proanthocyanidins of lotus seedpods possessed neuroprotection activity against amyloid-β-induced PC12 cells (10), extremely low frequency electromagnetic fields-induced primary cultured rat hippocampal neurons (66) and methyl mercuric chloride-induced neuron/astrocyte co-cultured cells (48). The activity was realized through inhibitions of oxidative stress and mitochondrial apoptotic pathway, and activation of CREB/BDNF signaling pathway. The proanthocyanidins raised levels of GSH, T-AOC and mitochondrial membrane potential, activities of SOD and GPX, protein expressions of p-CREB/CREB, BDNF, p-AKT/AKT, p-ERK/ERK, Bcl-xl, Bcl-2, SOD1/SOD2, Bcl-xl, Nrf2, HO-1, nuclear Nrf2, β-III-Tubulin, SYN and Arc and mRNA expression of BDNF, and down-regulated concentrations of LDH, MDA, Ca^2+^ and ROS, and protein expressions of Bad, Bax and caspase-3/-9 along with Bax/Bcl-2 ratio (10, 48, 66).

Animal experiment has demonstrated that proanthocyanidins of lotus seedpods reflected retinal protection against light exposure-induced rats through anti-oxidative stress, anti-apoptosis and neuroprotective effects, displaying as improving activities of SOD and GPX and mRNA and protein expressions of Bcl-2, and lowering retinal apoptosis, mRNA expressions of caspase-3, p53 and Bax, and protein expressions of pro-caspase-3, cleaved caspase-3, p53 and Bax (6). The proanthocyanidins exerted anti-insomnia effect to rats by regulating NO/ADMA/DDAH pathway, showing as increasing levels of 5-HT, GABA and NO, and protein and mRNA expressions of DDAH1, DDAH2 and nNOS, and decreasing levels of NE, Glu, ADMA and 8-isoprostane, in brain (67). Proanthocyanidins from lotus seedpods exhibited immunomodulatory effect against extremely low frequency electromagnetic fields-induced mice, and increased protein expressions of IL-2, IL-6, IL-10, INF-γ and Bcl-xl, and DNA contents, and decreased protein expressions of TNF-α and caspase-3/9 along with Bax/Bcl-2 ratio and apoptotic splenocytes in spleen (69). Otherwise, cell experiment has showed that proanthocyanidins from lotus seedpods possessed anti-cancer activity in HepG2 cells through inducing autophagy and ROS generation (68).

In addition, there are some connects between the different bioactivities of proanthocyanidins from lotus seedpods. The retinal protection of proanthocyanidins against light exposure-induced rats was realized partly through their antioxidation and neuroprotection activities (6). Ameliorating cognitive impairment action of proanthocyanidins was reported to be related to their antioxidation effects in D- galactose-, scopolamine-, alcohol and scopolamine, senescence-accelerated or extremely low frequency electromagnetic field exposure-induced mice (12, 52–54, 56, 61) as well as cognitively impaired aged rats (58). The neuroprotection of proanthocyanidins was associated with their antioxidation action in extremely low frequency electromagnetic field-induced primary cultured hippocampal neurons (66) and methyl mercuric chloride-induced neuron/astrocyte co-cultured cells (48).

### Biological activity of oligomeric procyanidins from lotus seedpods

Oligomeric procyanidin is another one of the mostly studied phytochemicals from lotus seedpods. They have been demonstrated to show antibacterial (15–17, 65), anti-glycative (7, 18, 19), ameliorating intestinal injury (70), anti-diabetes (20), antioxidation (5) and regulating lipid homeostasis (38) activities, as indicated in Figure 3C and Supplementary Table 1.

Oligomeric procyanidins of lotus seedpods showed antibacterial activity *in vitro* and *in vivo*. The oligomeric procyanidins had the minimal inhibitory concentrations on *Escherichia coli* K88ac, F18ac, 10899 and BL21 as 0.80, 1.20, 1.25, and 1.25 mg/mL, respectively (17, 65). Moreover, synergistic effect was observed between the oligomeric procyanidins and water-soluble *Poria cocos* polysaccharides or carboxymethyl pachyman in inhibitory effect on *Escherichia coli* (16, 17). The antibacterial mechanism of oligomeric procyanidins on *Escherichia coli* has been disclosed to be increments of extracellular alkaline phosphatase, ROS production, activities of SOD and CAT and mRNA expressions of sodA, soxR, oxyR and oxyS (15). Further study has indicated that the oligomeric procyanidins reflected antibacterial activity against high-lactose diet-induced mice by enhancing abundances of *Lactobacillus* and *Bifidobacterium*, and lessening populations of *Escherichia coli* and *Enterococcus* in feces (15). On the other hand, the oligomeric procyanidins ameliorated intestinal injury against enterotoxigenic *Escherichia coli*-infected diarrhea mice (70). This effect was related to the modulation of TLR4-MAPK signaling pathway, reflecting as increasing protein and mRNA expressions of ZO-1, claudin-1 and occludin, and decreasing protein expressions of p-p38, p-JNK1/2 and p-ERK1/2 and RNA expressions of TNF-α, IL-8, IL-1β, IL-6, CD14, TLR4, p38 and NF-κB in jejunum/ileum.

Oligomeric procyanidins from lotus seedpods exerted anti-glycative activity on Caco-2 cells treated with digestive fluid (19), high-AGEs diet-induced mice (7) and high-fat diet-induced rats (18). RAGE-p38MAPK-NF-κB signaling pathway was involved in their activities. In the animal experiments, the oligomeric procyanidins decreased protein expressions of NF-κB, p38MAPK, p-p38MAPK, RAGE and p65NF-κB, and mRNA expressions of TNF-α, IL-6, NADPH, COX and RAGE in liver of mice or rats induced by high-AGEs diet or high-fat diet (7, 18). In the cell experiment, the oligomeric procyanidins reduced protein expressions of RAGE, p-p38MAPK, p65NF-κB and p-p65NF-κB, and mRNA expressions of NADPH, TNF-α, IL-6, ICAM-1 and VCAM-1 of Caco-2 cells treated with digestive fluid (19). Moreover, absorption and metabolism of oligomeric procyanidins from lotus seedpods have been investigated in the study of Wu et al. (72). Eight metabolites, including (+)-catechin, caffeic acid, syringic acid, 3-hydroxybenzoic acid, 3-hydroxyphenylacetic acid, 3-hydroxyphenylpropionic acid, ferulic acid and *m*-coumaric acid, have been detected in in urine of rats after 24 h post-administration of 300 mg/kg body weight of the oligomeric procyanidins. Among them, (+)-catechin had much better inhibition activity on AGE formation and methylglyoxal scavenging effect, while syringic acid showed the best scavenging ability on DPPH radical. Furthermore, Wu et al. (49) have compared the inhibitory effects of oligomeric procyanidin from lotus seedpods and its three main monomers [(+)-catechin, (-)-epicatechin and (-)-epigallocatechin gallate] on releases of AGE and CML formation in simulated gastrointestinal digestion, for studying the structure-activity relationship. The results indicated that (-)-epigallocatechin gallate exhibited the strongest activities.

Animal experiment has shown that oligomeric procyanidins from lotus seedpods possessed anti-diabetes action against streptozotocin-induced mice by attenuating mTOR signaling and enhancing glucose homeostasis (20). This action was related to enhancement of protein expressions of GLUT2, GK, p-AKT, UCP-1 and GLUT4 and mRNA expressions of HK II, PFK and PK, and reduction of protein expressions of mTOR, p66^Shc^, PKCβ, FoxO1a, p-FoxO1a and GLUT1 and mRNA expressions of PEPCK, G-6-Pase, SREBP-1c, ACL, ACC1, FAS, SCD1 and S6K1, in liver, skeletal muscle, white adipose tissue and/or brown adipose tissue. Chemical assays have revealed that the oligomeric procyanidins had antioxidation action in scavenging ⋅OH, O^2–^ and H_2_O_2_ radicals (5). Animal investigation has suggested that the oligomeric procyanidins regulated the lipid profile of high fat/sucrose diet-induced rats by suppressing the lipogenesis-related gene expressions, such as SREBP-1c, FAS, ACC1, PPARγ and CD36, and elevating phase II drug metabolism enzyme SULT2B1b gene expression (38).

Additionally, some connects between the different bioactivities of oligomeric procyanidin could be found. Wu et al. (7, 18, 19, 72) have argued that anti-glycative activity of oligomeric procyanidins from lotus seedpods is positively correlated to their antioxidant capacities. The study taken by Tang et al. (70) has indicated that the antioxidant mechanism involved the ameliorating intestinal injury activity of oligomeric procyanidins from lotus seedpods on *Enterotoxigenic Escherichia coli* infected diarrhea mice.

### Biological activity of others phytochemicals from lotus seedpods

Others phytochemicals like β-sitosterol, quercetin, kaempferol and polysaccharides from lotus seedpods have been indicated to have anti-tyrosinase (46, 47), anti-inflammatory (71), antioxidation (4) and α-glucosidase inhibitory (4) effects. β-sitosterol, quercetin 3-*O*-β-D-galactopyranoside and kaempferol 3-*O*-β-D-glucopyranoside from lotus seedpods have been determined to be tyrosinase inhibitors (46, 47) as shown in Supplementary Table 1. A (E)-9-Octadecenoic acid ethyl ester from lotus seedpods showed anti-inflammatory effect against LPS-induced RAW264.7 cells, and the effect was realized through regulating MAPKs and NF-κB signaling pathways, which displaying as up-regulations of NF-κB nuclear translocation, protein expressions of ERK, p38 and JNK, and protein and mRNA expressions of iNOS and COX2 (71). Flavonol glycosides fractionated from extracts of lotus seedpods, namely hyperoside, isoquercitrin, quercetin-3-*O*-β-D-glucuronide, isorhamnetin-3-*O*-β-D-galactoside and syringetin-3-*O*-β-D-glucoside, appeared scavenging activities on ABTS and DPPH radicals (35). Our previous study has indicated that water-extracted polysaccharides from dried lotus seedpods possessed good scavenging effects on ABTS, DPPH and ⋅OH radicals, and an obvious inhibitory effect on α-glucosidase activity (4).

## Applications of raw material, extracts and phytochemicals of lotus seedpods

### Applications of lotus seedpods

Lotus seedpods can be applied as sources for biochar (21, 73–76) and biomass material (22, 77–80).

As a source of biochar, lotus seedpods can be used to constitute sensor (74), absorber (21, 73, 75, 81) and detector (76). A portable, flexible, outdoor and inexpensive sensing platform for hyperin has been established by lotus seedpods biochar and molybdenum disulfide, using a green co-hydrothermal approach (74). Lotus seedpod-derived biochar can be used for producing available and effective biosorbents for cadmium (73), methylene blue (81) and 17 β-estradiol (21), along with Co_3_O_4_ microwave absorbent (75). Otherwise, a carbon quantum dots was synthesized on the basis of lotus seedpod by hydrothermal synthesis method, which could be utilized for Fe(III) detection (76).

In terms of using for biomass material, lotus seedpod is high-stable electrode material for supercapacitors (22, 78, 80). Meanwhile, lotus seedpod-derived hard carbon with hierarchical porous structure is a stable anode for sodium-ion batteries (79). Otherwise, an efficient metal-free catalyst derived from lotus seedpod exhibited excellent oxygen reduction reaction (77).

### Applications of lotus seedpods extracts and phytochemicals

Lotus seedpod extracts (8, 82), proanthocyanidin (83, 84) and oligomeric procyanidin (5, 19, 23, 43, 44, 49, 72, 85, 86) can be used in food industry. Lotus seedpod extracts can inhibit lipid oxidation. 80% ethanol extract and water extract reduced the acid value, peroxide value and TBARS level of lard (82). 50% ethanol extract decreased the peroxidation level of linoleic acid (8). Moreover, conjugate complexes produced by lotus seedpod proanthocyanin and whey protein have potential applications in emulsions (83, 84). Lotus seedpod proanthocyanidin-whey protein complexes improved the chemical stability of β-carotene nanoemulsions (84). Lotus seedpod proanthocyanidin was grafted to whey protein isolate for creating nature-derived antioxidant emulsifiers, which had good DPPH radical scavenging activity and ferric reducing ability (83).

Inhibition of AGEs formation is an important application for lotus seedpod oligomeric procyanidin in food systems, such as bovine serum albumin-glucose (43, 72), lactose-lysine (44) and yogurt (86) systems. Meanwhile, addition of lotus seedpod oligomeric procyanidin increased the growth of *Lactobacillus plantarum*, titratable acidity, DPPH scavenging effect, solid-like properties, hardness, adhesiveness, gumminess and chewiness of yogurt (86). Moreover, lotus seedpod oligomeric procyanidin and its three monomers including catechin, epicatechin and epigallocatechin gallate could inhibit AGEs release from glycated casein during gastrointestinal digestion (49). At the same time, lotus seedpod oligomeric procyanidin and catechin could also enhance the scavenging effects of digestive fluid on DPPH, OH and ABTS radicals and its ferric reducing ability (19). On the other hand, lotus seedpod oligomeric procyanidin is beneficial to the storage and process of food. Lotus seedpod oligomeric procyanidin could decline the peroxide values of lard or soybean oil (5). Lotus seedpod oligomeric procyanidin possessed inhibitory effect on the retrogradation property of rice starch (23).

Beside, oligomeric procyanidin extracted from lotus seedpod has been used to dye the tussah silk fabric (87).

## Conclusion and perspectives

Lotus seedpod is a promising food and medicine source. Extracts of lotus seedpods obtained by non-organic and organic solvents exerted antioxidation, anti-cancer, anti-melanogenic, anti-inflammatory, anti-irradiation, cardioprotection and hepatoprotection activities. Meanwhile, extraction solvents and methods have influences on the activities. Diversity phytochemicals are responsible for these bioactivities, such as proanthocyanidins, oligomeric procyanidins, flavonoids, alkaloids, terpenoids, organic acids, steroids, esters and others. Some of phytochemicals have been well-identified by modern analytical techniques, and chemical structures of some phytochemicals have been proposed. Moreover, chemical assays as well as cell and animal experiments have demonstrated that phytochemicals (especially proanthocyanidins and oligomeric procyanidins) from lotus seedpods exhibited broad-spectrum biological activities, including ameliorating cognitive impairment, antioxidation, antibacterial, anti-glycative, neuroprotection, anti-tyrosinase, retinal protection, anti-insomnia, anti-cancer, immunomodulatory, ameliorating intestinal injury, anti-diabetes, regulating lipid homeostasis, anti-inflammatory and α-glucosidase inhibitory effects. Furthermore, raw material, extracts and phytochemicals of lotus seedpods have been applied as sources for biochar and biomass material, in food industry, and as dye. In contrast, other parts of lotus like seeds, stems, leaves, flowers, epicarps, plumules, stamens, petals and rhizomes have been reported to contain phytochemicals included flavonoids, glycosides, alkaloids, monosaccharides, essential oils, organic acids, chlorophylls, sesquiterpenoids, steroids, sapogenins, etc (2). Biological activities of these parts of lotus have been summarized as anti-obesity, antioxidant, cardiovascular, hepatoprotective, hypoglycemic, antimicrobial, lipolytic, anti-amnesic, anti-inflammatory, antithrombotic, anti-proliferative, memory-improving, sedative, immunoregulatory and antiviral activities (2). These parts of lotus can be applied in health food industry and medicine (2). Therefore, to lotus industry, lotus seedpods are worth exploiting for acquisition of novel phytochemicals, enrichment of more biological activities and new attempts of industrial applications.

However, there are some issues should be resolved in future: (1) the phytochemicals from lotus seedpod is mainly prepared using conventional solvent method, more attempts should be made to novel means; (2) although many phytochemicals with small molecular weight have been isolated and identified from lotus seedpods, the exploitations of those (like proteins and polysaccharides) with large molecular weight have been rarely done; (3) there is little information on digestion, absorption, distribution, structure-activity relationship, metabolism of phytochemicals from lotus seedpod, which need to be disclose with more work; (4) the current researches on biological activities of phytochemicals from lotus seedpods mostly foucused on proanthocyanidins and oligomeric procyanidin, the biological activities of other phytochemicals from lotus seedpod; (5) biological activities of extracts and phytochemicals from lotus seedpods have been indicated in chemical assays as well as cell and animal experiments, but have not been demonstrated by clinical research; (6) the action mechanisms about the relevant activities of extracts and phytochemicals from lotus seedpods are unclear as they have been preliminarily explored.

## Author contributions

Y-FW: conceptualization, investigation, visualization, and writing—original draft. Z-CS and JL: investigation and visualization. TL, J-LS, and X-FL: investigation. Y-PL: project administration and funding acquisition. WZ: writing—review and editing. QZ: funding acquisition. X-YW: project administration, writing—review and editing, supervision, and funding acquisition. All authors contributed to the article and approved the submitted version.

## Abbreviations

DPPH: 1,1-diphenyl-2-picrylhydrazyl
ABTS: 2,2′-azino-bis-(3-ethylbenzthiazoline-6-sulfonic acid)
OH: hydroxyl radical
O^2–^: superoxide anion
MDA: malondialdehyde
TBARS: thiobarbituric acid reactive substances
SOD: superoxide dismutase
CAT: catalase
GPX: glutathione peroxidase
GSH: glutathione
LPO: lipid peroxidation
ROS: reactive oxygen species
NO: nitric oxide
Ang II: angiotensin II
PKC: protein kinase C
p-PKC: phosphorylation PKC
ERK: extracellular-signal-regulated kinase
p-ERK: phosphorylation ERK
NADPH: nicotinamide adenine dinucleotide phosphate
NOX: NADPH oxidase
AT1R: type I angiotensin receptor
RAGE: receptor for advanced glycation end products
MAPK: mitogen-activated protein kinase
TRP: tyrosinase-related protein 1
PKA: protein kinase A
CREB: cAMP-response element binding protein
p-CREB: phosphorylation CREB
MITF: melanocyte inducing transcription factor
JNK: c-Jun N terminal kinase
p-JNK: phosphorylation JNK
AchE: acetylcholinesterase
MC1R: melanocortin-1 receptor
PARP: poly adenosine diphosphoribose polymerase
Axl: tyrosine kinase receptor
IL-1β: interleukin-1β
IL-2: interleukin-2
IL-6: interleukin-6
IL-8: interleukin-8
IL-10: interleukin-10
NF-κB: nuclear factor kappa B
p-NF-κB: phosphorylation NF-κB
Nrf2: nuclear respiratory factor 2
HO-1: heme oxygenase-1
LPS: lipopolysaccharide
TNF-α: tumor necrosis factor-α
COX-2: cyclooxygenase-2
NOS: nitric oxide synthase
iNOS: inducible NOS
nNOS: neuronal NOS
tNOS: total NOS
TLR4: toll-like receptor 4
MyD88: myeloid differentiation primary response gene 88
ZO-1: zonula occludens-1
CD14: cluster of differential 14
AGEs: advanced glycation end products
MAO-B: monoamine oxidase B
Aβ: β-Amyloid peptide
CaMKII: calcium/calmodulin-dependent protein kinase II
BDNF: brain-derived neurotrophic factor
DAG: diacylglycerol
IP3: ins(1,4,5)P_3_
PP2B: protein phosphatase 2B
T-AOC: total antioxidant capacity
ADMA: asymmetric dimethylarginine
DDAH: dimethylarginine dimethylaminohydrolase
5-HT: 5-hydroxytryptamine
GABA: γ-aminobutyric acid
Glu: glutamic acid
NE: noradrenaline
mTOR: mammalian target of rapamycin
GK: glucokinase
FoxO1a: forkhead box O1
p-FoxO1a: phosphorylation FoxO1a
SREBP-1c: sterol regulatory element-binding protein 1c
G-6-Pase: glucose-6-phosphatase
ACC1: acetyl coenzyme A carboxylase 1
ACL: ATP-citrate lyase
FAS: fatty acid synthase
SCD1: stearoyl CoA desaturase 1
S6K1: S6 Kinase 1
PEPCK: phosphoenolpyruvate carboxykinase
PFK: phosphofructokinase
PK: pyruvate kinase
HK II: hexokinase II
UCP-1: uncoupling protein 1
GR: glutathione reductase
GST: glutathione-*S*-transferase
LDH: lactate dehydrogenase
AKT: protein kinase B
p-AKT: phosphorylation AKT
Ach: acetylcholine
NR2B: NMDA receptor 2B
ASK: apoptosis signal regulating kinase
CML: N^ϵ^-(carboxymethyl) lysine
FFA: free fatty acids
TC: cholesterol
TG: triglyceride
AST: aspartate aminotransferase
ALT: alanine aminotransferase
ALP: alkaline phosphatase
HDL-C: high-density lipoprotein cholesterol
GRAC: glutathione reductase
ICAM-1: intercellular adhesion molecule-1
LDL: low-density lipoprotein
VCAM-1: vascular cell adhesion molecule-1
LC3: light chain 3
Atg5/12: autophagy elongation complex
PI3K: phosphoinositide 3-kinase
Beclin-1: the mammalian ortholog of yeast ATG6
Bcl-2: B-cell lymphoma-2
Bax: Bcl2-associated X
Bad: Bcl2 associated death promoter
p-Bad: phosphorylation Bad
IκB: inhibitor of NF-κB
IKK: IκB kinase
p-IκB: phosphorylation IκB
γ-H2AX: phosphorylated histone family 2A variant
NFATc-1: nuclear factor of activated T cells cytoplasmic 1
ANP: atrial natriuretic peptide
BNP: B-type natriuretic peptide
MLC2: myosin light chain 2
ET-1: endothelin-1
SYN: synaptophysin
Arc: activity-regulated cytoskeleton−associated protein
INF-γ: gamma-interferon
GLUT: glucose transporter
PPAR: peroxisome proliferator-activated receptors
SULT2B1b: sulfotransferase 2B1b.
