# Supplementary material for: Phytochemicals, biological activity, and industrial application of lotus seedpod (Receptaculum Nelumbinis): A review
Source: Front Nutr. 2022 Oct 4;9:1022794. doi: 10.3389/fnut.2022.1022794 (PMC9577462; doi:10.3389/fnut.2022.1022794)
Supplement: Supplementary file 1 [file Table_1.docx]

**Table S1** Biological activities of extracts and phytochemicals from lotus seedpods (*Receptaculum Nelumbinis*)

| Name | Health-promoting property | Target model | Changes of biochemical index | Reference |
| --- | --- | --- | --- | --- |
| **Different extracts** | | | | |
| 80% ethanol, *n*-hexane, chloroform, ethyl acetate, butanol and water extracts | Antioxidation | Scavenging free radical assay; ferric reducing ability assay | ↑ Scavenging activities of DPPH and ABTS radicals; ferric reducing ability | (29) |
| 75% ethanol extract | Antioxidation | Scavenging free radical assay | ↑ Scavenging activities of DPPH and ABTS radicals | (35) |
| 70% ethanol extract | Antioxidation | Scavenging free radical assay; ferric reducing ability assay | ↑ Scavenging activities of DPPH, •OH, O^2-^ and H_2_O_2_ radicals; ferric reducing ability | (34) |
|  |  | H_2_O_2_-induced RAW264.7 cells | ↑ Cell viability;  ↓ MDA level |  |
| Water extract | Antioxidation | H_2_O_2_-induced RIN-m5F cells | ↑ Cell viability and autophagy; insulin level; protein expressions of LC3II, Atg5/12, p62, class III PI3K and Beclin-1 as well as p-Bad/Bad;  ↓ Levels of TBARS and ROS; cell apoptosis; protein expressions of active-caspase-3 and cleavage PARP-1 as well as Bax/Bcl-2 | (9) |
| Glycerol extract | Antioxidation | Scavenging free radical assay; ferric reducing ability assay | ↑ Scavenging activities of DPPH and ABTS radicals; ferric reducing ability | (13,36) |
| 50% ethanol extract | Antioxidation | Scavenging free radical assay; ferric reducing ability and metal ion chelating activity assays | ↑ Scavenging activities of DPPH, •OH, O^2-^ and ABTS radicals; ferric reducing ability; metal ion chelating activity | (8) |
|  | Anti-cancer effect | HepG2 and LNcap cells | ↓ Cell proliferation |  |
| Subcritical water or water extract | Antioxidation | Scavenging free radical assay | ↑ Scavenging activities of DPPH, ABTS and NO_2_^-^ radicals; ferric reducing ability | (14) |
|  | Anti-cancer effect | HepG2 cells | ↓ Cell proliferation |  |
| 70% ethanol extract | Anti-melanogenic effect | α-melanocyte stimulating hormone-induced B16F10 cells | ↓ Melanin synthesis; activity and protein expression of tyrosinase | (33) |
| Water extract | Anti-melanogenic effect | α-melanocyte stimulating hormone-induced B16F10 cells | ↓ Melanin synthesis; tyrosinase activity; protein and mRNA expressions of tyrosinase, TRP-1 and MC1R; cAMP level; protein expression of MITF and protein expression ratios of p-PKA/PKA, p-p38/p38 and p-CREB/CREB | (27) |
|  |  | UVB-induced mice | ↓ Melanin content, protein expressions of tyrosinase, TRP-1 and TRP-2 as well as p-PKA/PKA and p-p38/p38 in ears |  |
| Acetone-water extract | Anti-irradiation effect | ^60^Co irradiation-induced mice | ↑ Survival time; activities of SOD, CAT and GPX in liver; levels of white blood cells, red blood cells, platelets and hemoglobin; spleen weight and index;  ↓ LPO level in liver; chromosomal aberrations in the bone marrow | (37) |
| 70% ethanol extract | Anti-cancer effect | A549 and H460 cells | ↑ Cell apoptosis; protein expressions of cleavage PARP and γ-H2AX;  ↓ Cell proliferation; PARP protein expression; protein and mRNA expressions of Axl | (32) |
| Ethanol extract | Anti-inflammatory effect | LPS-induced RAW264.7 cells | ↑ Cell viability; protein expressions of Nrf2 and HO-1;  ↓ Productions of NO and ROS; protein expressions of iNOS, COX-2, p65 and p-p65 as well as protein expression ratios of p-p38/p38, p-ERK/ERK and p-JNK/JNK | (31) |
| Water extract | Anti-inflammatory effect | LPS-induced HepG2 cells | ↓ Levels of TNF-α and IL-6; mRNA expressions of IL-6, COX-2 and iNOS; protein expressions of COX-2, iNOS, NF-κB, IKK, TLR4 and MyD88 as well as p-IκB/IκB and p-p38/p38; | (28) |
|  |  | LPS-induced mice | ↑ GSH content and SOD activity in liver;  ↓ Levels of AST and ALT in serum; levels of TNFα and IL-6 in liver; protein expressions of COX-2, iNOS, NF-κB and IKK as well as p-IκB/IκB and p-p38/p38 in liver |  |
| 100% methanol extract | Cardioprotection | Ang II-induced H9c2 cells | ↑ LDH activity;  ↓ Cell size; protein expressions of NFATc-1, ANP, BNP, MLC2, NOX2, NOX4, AT1R, RAGE, PKC, p-PKC and p-ERK1/2 as well as p-NF-κB/NF-κB; protein synthesis; ROS and HMGB1 level | (30) |
| Water extract | Hepatoprotection | Oleic acid-induced HepG2 cells | ↑ Cell viability; Bcl-2 protein expression;  ↓ Lipid accumulation; ROS production; cell autophagy; protein expressions of Atg5/12, active-caspase-3/8/9, cleaved PARP and Bax as well as Bax/Bcl-2 and LC3-II/LC3-I; cell apoptosis; mitochondrial membrane depolarization | (26) |
| **Proanthocyanidins** | Ameliorating cognitive impairment | D-Galactose-induced mice | ↑ Learning and memory abilities of mice; activities of SOD and GPX in brain;  ↓ Levels of Aβ_1-42_, NO and MDA, and activities of AchE, MAO-B, tNOS and nNOS in brain; nerve cell apoptosis and p53 protein expression in hippocampus | (12) |
|  | Ameliorating cognitive impairment | Senescence-accelerated mice | ↑ Learning and memory abilities of mice; GSH level, and activities of SOD and GPX in brain and serum;  ↓ Levels of NO and MDA in brain and serum; nNOS activity in brain; total NOS activity in serum | (61) |
|  | Ameliorating cognitive impairment | Aged rats | ↓ NO level, activities of tNOS and iNOS, and protein and mRNA expressions of iNOS in hippocampus and cerebral cortex | (60) |
|  |  |  | ↑ Protein expressions of p-CREB and BDNF as well as p-ERK42/ERK42 and p-ERK44/ERK44 in hippocampus; BDNF mRNA expression in hippocampus | (59) |
|  |  |  | ↑ Learning and memory abilities of rats; levels of GSH, T-AOC, AchE and Ach, and activities of CAT and GPX in hippocampus and cerebral cortex;  ↓ MDA levels in hippocampus and cerebral cortex | (58) |
|  | Antioxidation |  | ↑ Activities of SOD, CAT and GPX, and GSH level in serum, heart, liver, kidney, lung or muscle;  ↓ TBARS contents in serum, heart, liver, kidney, lung or muscle | (64) |
|  | Ameliorating cognitive impairment | Extremely low frequency electromagnetic fields-induced mice | ↑ Protein expressions of CaMKII, PKCα and BDNF in hippocampus;  ↓ Protein expressions of Gi, PKA, PKCβ and PP2B, Ca^2+^ content and concentrations of IP3 and DAG in hippocampus | (11) |
|  |  |  | ↑ Protein expressions of p-ERK1/2 in hippocampus;  ↓ Concentrations of glutamate, GABA and [Ca^2+^]_i_, and protein expressions of ASK1, NR2B, p-CREB and p-JNK1/2 in hippocampus | (57) |
|  |  |  | ↑ Learning and memory abilities of mice; activities of SOD, CAT and GPX in serum and hippocampus; pyramidal cell numbers in hippocampus;  ↓ ROS level in hippocampus; levels of MDA and NO, and NOS activity in serum and hippocampus | (56) |
|  | Antioxidation |  | ↑ Activities of SOD, CAT, GPX, GR and GST in serum and cerebral cortex;  ↓ MDA level in serum and cerebral cortex | (63) |
|  | Ameliorating cognitive impairment | Scopolamine-induced mice | ↑ Learning and memory abilities of mice; T-AOC level and activities of SOD and GPX in brain, serum and colon;  ↓ MDA level in brain, serum and colon; activities of MPO, AchE, tNOS and nNOS in brain; nNOS mRNA expression in brain | (53) |
|  |  |  | ↑ Learning and memory abilities of mice; activities of SOD and GPX in brain;  ↓ Levels of MAO-B and AchE, and activities of tNOS and nNOS in brain | (54) |
|  |  |  | ↑ Learning and memory abilities of mice;  ↓ Levels of AchE and nNOS in brain | (55) |
|  | Ameliorating cognitive impairment | alcohol- or scopolamine-induced mice | ↑ Learning and memory abilities of mice; T-AOC level and activities of SOD and GPX in brains;  ↓ Levels of MDA, MAO-B and AchE in brains; serum iNOS activity; NO level and iNOS activity in hippocampus | (52) |
|  | Neuroprotection | Amyloid-β-induced PC12 cells | ↑ Cell viability; SOD activity; protein and mRNA expressions of BDNF; protein expression ratios of p-CREB/CREB, p-AKT/AKT and p-ERK/ERK;  ↓ Cell apoptosis, concentrations of LDH and MDA | (10) |
|  | Antioxidation | DPPH and ABTS free radicals; ferric reducing ability assay | ↑ Scavenging activities of DPPH and ABTS radicals; ferric reducing ability | (62) |
|  |  | H_2_O_2_-induced HUVECs | ↑ Cell viability; activities of SOD and GPX; NO level;  ↓ Levels of MDA and ET-1 |  |
|  | Neuroprotection | Extremely low frequency electromagnetic fields-induced primary cultured rat hippocampal neurons | ↑ Cell viability; SOD activity; cells accumulation in G0/G1 phase; protein expressions of Bcl-xl and Bcl-2; mitochondrial membrane potential;  ↓ Levels of MDA, Ca^2+^ and ROS; DNA damage; cells accumulation in S phase; cell apoptosis and necrosis; protein expressions of Bad and Bax along with Bax/Bcl-2 | (66) |
|  | Neuroprotection | Methyl mercuric chloride-induced neuron/astrocyte co-cultured cells | ↑ Cell viability; mitochondrial membrane potential; levels of GSH and T-AOC; GPX activity; protein expressions of SOD1/SOD2, Bcl-xl, Nrf2, HO-1, nuclear Nrf2, β-Ⅲ-Tubulin, SYN and Arc;  ↓ Levels of LDH and Ca^2+^; protein expressions of caspase-3/-9 as well as Bax/Bcl-2 | (48) |
|  | Antibacterial activity | *Escherichia coli* | ↑ Extracellular alkaline phosphatase; ROS production; activities of SOD and CAT; mRNA expressions of sodA, soxR, oxyR and oxyS;  ↓ Bacterial growth | (15) |
|  |  | High-lactose diet-induced mice | ↑ *Lactobacillus* and *Bifidobacterium* in faeces;  ↓ *Escherichia coli* and *Enterococcus* in faeces |  |
|  | Retinal protection | Light exposure-induced rats | ↑ ONL thickness; activities of SOD and GPX; Bcl-2 mRNA and protein expressions;  ↓ Levels of MDA, NO and NOS in serum; retinal apoptosis; mRNA expressions of caspase-3, p53 and Bax; protein expressions of pro-caspase-3, cleaved caspase-3, p53 and Bax | (6) |
|  | Anti-insomnia | Insomnia rats | ↑ Sleep duration; levels of 5-HT, GABA and NO, and protein and mRNA expressions of DDAH1, DDAH2 and nNOS in brain;  ↓ Sleep latency; walking time and forelimb lifting-up frequency; NE, Glu, ADMA and 8-isoprostane levels in brain | (67) |
|  | Anti-cancer | HepG2 cells | ↑ Cell autophagy; protein expressions of LC3-II; ROS generation; DNA damage; S cycle arrest  ↓ Cell viability; mitochondrial membrane potential; cyclin A protein expression | (68) |
|  | Immunomodulatory effect | Extremely low frequency electromagnetic fields-induced mice | ↑ Spleen index; number and percentage of lymphocytes in blood; WBC number, RBC number and hemoglobin in blood; protein expressions of IL-2, IL-6, IL-10, INF-γ and Bcl-xl, and DNA contents in spleen;  ↓ Protein expressions of TNF-αand caspase-3/9 as well as Bax/Bcl-2, apoptotic splenocytes, and G0/G1 phase cycle arrest in spleen | (69) |
| **Oligomeric procyanidins** | Antibacterial activity | *Escherichia coli* K88ac and F18ac | ↓ Bacterial growth | (65) |
|  | Antibacterial activity | *Escherichia coli* 10899 | ↓ Bacterial growth | (16) |
|  | Antibacterial activity | *Escherichia coli* 10899 and BL21 | ↓ Bacterial growth | (17) |
|  | Ameliorating intestinal injury | Enterotoxigenic *Escherichia coli*-infected mice | ↑ SOD activity, GSH level, protein and mRNA expressions of ZO-1, claudin-1 and occludin in jejunum/ileum; concentrations of Na^+^ and Cl^-^ in serum;  ↓ Diarrhea signs; white blood cells numbers and percentage of neutrophil leukocytes; MDA level in jejunum/ileum; DAO activity in serum; protein expressions of p-p38, p-JNK1/2 and p-ERK1/2 in jejunum; mRNA expressions of TNF-α, IL-8, IL-1β, IL-6, CD14, TLR4, p38 and NF-κB | (70) |
|  | Anti-diabetes | Streptozotocin-induced mice | ↑ Glucose homeostasis, levels of HDL and insulin; protein expressions of GLUT2, GK and p-AKT; mRNA expressions of PFK and PK in liver and skeletal muscle; protein expressions of GLUT2 and GK in skeletal muscle; mRNA expressions of HKⅡ, PFK and PK in white adipose tissue; protein expressions of UCP-1 and GLUT4 in brown adipose tissue;  ↓ Blood glucose; levels of TG, TC, LDL, FFA, endotoxin, GHbA1c, AST and ALT; protein expressions of mTOR, p66^Shc^, PKCβ, FoxO1a and p-FoxO1a, and mRNA expressions of PEPCK, G-6-Pase, SREBP-1c, ACL, ACC1, FAS, SCD1 and S6K1 in liver; protein expressions of mTOR and p66^Shc^ in skeletal muscle and white adipose tissue; SREBP-1c mRNA expression in white adipose tissue; GLUT1 protein expression in brown adipose tissue | (20) |
|  | Anti-glycative activity | High-AGEs diet-induced mice | ↑ Levels of T-AOC and GSH, and activities of SOD and CAT in liver;  ↓ Contents of AGEs, CML, MDA and LPS, protein expressions of NF-κB, p38MAPK, p-p38MAPK and RAGE, and mRNA expressions of TNF-α and IL-6 in liver | (7) |
|  | Anti-glycative activity | High-fat diet-induced rats | ↑ Levels of HDL-C and T-AOC, and activities of SOD and GPX in serum;  ↓ Levels of TG, TC, FFA, AST, ALT, ALP, MDA, GRAC and NO in serum; levels of ROS, AGEs, CML, TNF-α, IL-1, IL-6, IL-8, ICAM-1, VCAM-1, MCP-1 and E-selectin, mRNA expressions of NADPH, COX and RAGE, and protein expressions of p38MAPK, p-p38MAPK and p65NF-κB in liver | (18) |
|  | Anti-glycative activity | Caco-2 cells treated with digestive fluid | ↑ Cell viability; GSH level; CAT activity;  ↓ Cell apoptosis; levels of ROS, TNF-α and IL-6; protein expressions of RAGE, p-p38MAPK, p65NF-κB and p-p65NF-κB; mRNA expressions of NADPH, TNF-α, IL-6, ICAM-1 and VCAM-1 | (19) |
|  | Antioxidation | Free radicals scavenging assay | ↑ Scavenging activities of •OH, O^2-^ and H_2_O_2_ radicals | (5) |
|  | Regulating lipid homeostasis | High fat/sucrose diet-induced rats | ↑ HDL level in serum; SULT2B1b mRNA expression in liver;  ↓ Levels of TC and TG in serum; mRNA expressions of SREBP-1c, FAS, ACC1, PPARγ and CD36 in liver | (38) |
| **Others** | | | | |
| β-sitosterol, quercetin 3-O-β-D-galactopyranoside, kaempferol 3-O-β-D-glucopyranoside, etc. | Anti-tyrosinase activity | Tyrosinase inhibitory assay | ↓ Tyrosinase activity | (46,47) |
| (E)-9-Octadecenoic acid ethyl ester | Anti-inflammatory effect | LPS-induced RAW264.7 cells | ↑ Cell viability;  ↓ Productions of NO and PGE2; protein and mRNA expressions of iNOS and COX2; TNF-α level; protein expressions of ERK, p38 and JNK; nuclear translocation of NF-κB | (71) |
| Flavonol glycosides | Antioxidation | Free radicals scavenging assay | ↑ Scavenging activities of ABTS and DPPH radicals | (35) |
| Polysaccharides | Antioxidation | Free radicals scavenging assay | ↑ Scavenging activities of ABTS, DPPH and •OH radicals | (4) |
|  | α‐glucosidase inhibitory effect | α‐glucosidase inhibitory assay | ↓ α‐Glucosidase activity |  |

↑ Increasing or promoting; ↓ Decreasing or suppressing.
